# METTL3-Mediated LncRNA *EN_42575* m6A Modification Alleviates CPB2 Toxin-Induced Damage in IPEC-J2 Cells

**DOI:** 10.3390/ijms24065725

**Published:** 2023-03-16

**Authors:** Jiaojiao Yang, Qiaoli Yang, Xiaoyu Huang, Zunqiang Yan, Pengfei Wang, Xiaoli Gao, Jie Li, Shuangbao Gun

**Affiliations:** 1 College of Animal Science and Technology, Gansu Agricultural University, Lanzhou 730070, China; 2 Gansu Research Center for Swine Production Engineering and Technology, Lanzhou 730070, China

**Keywords:** m6A, lncRNA *EN_42575*, CPB2 toxin, IPEC-J2 cell, METTL3

## Abstract

Long non-coding RNAs (lncRNAs) modified by n6-methyladenosine (m6A) have been implicated in the development and progression of several diseases. However, the mechanism responsible for the role of m6A-modified lncRNAs in *Clostridium perfringens* type C piglet diarrhea has remained largely unknown. We previously developed an in vitro model of CPB2 toxin-induced piglet diarrhea in IPEC-J2 cells. In addition, we previously performed RNA immunoprecipitation sequencing (MeRIP-seq), which demonstrated lncRNA *EN_42575* as one of the most regulated m6A-modified lncRNAs in CPB2 toxin-exposed IPEC-J2 cells. In this study, we used MeRIP-qPCR, FISH, EdU, and RNA pull-down assays to determine the function of lncRNA *EN_42575* in CPB2 toxin-exposed IPEC-J2 cells. LncRNA *EN_42575* was significantly downregulated at different time points in CPB2 toxin-treated cells. Functionally, lncRNA *EN_42575* overexpression reduced cytotoxicity, promoted cell proliferation, and inhibited apoptosis and oxidative damage, whereas the knockdown of lncRNA *EN_42575* reversed these results. Furthermore, the dual-luciferase analysis revealed that METTL3 regulated lncRNA *EN_42575* expression in an m6A-dependent manner. In conclusion, METTL3-mediated lncRNA *EN_42575* exerted a regulatory effect on IPEC-J2 cells exposed to CPB2 toxins. These findings offer novel perspectives to further investigate the function of m6A-modified lncRNAs in piglet diarrhea.

## 1. Introduction

N6-methyladenosine (m6A) modifications are the most abundant internal RNA modifications in eukaryotes that have been reported to regulate RNAs [1]. The m6A modification is a dynamic and reversible process, which is regulated primarily by three classes of methylation-related enzymes [2]. There are various methylation transferases involved in m6A formation, such as methyltransferase-like 3 (METTL3), methyltransferase-like 14 (METTL14), Wilms’ tumor 1-associated protein (WTAP), RNA-binding motif protein 15 (RBM15), methyltransferase-like protein 16 (METTL16), and vir-like m6A methyltransferase-associated protein (VIRMA). METTL3, METTL14, and WTAP form a complex, which is the core component of m6A methylesterase and is crucial for its function [3]. The two main types of demethylases are obesity-associated protein (FTO) and alpha-ketoglutarate-dependent dioxygenase (ALKB) homolog 5 (ALKBH5) [4]. Several m6A-binding proteins have been reported, including the YT521-B homologous domain (YTH) family (YTHDC1, YTHDC2, YTHDF1, YTHDF2, and YTHDF3); the heterogeneous nuclear ribonucleoprotein (HNRNP) family (HNRNPA2B1, HNRNPC, and HNRPG); insulin-like growth factor 2 mRNA-binding protein 1/2/3 (IGF2BP1/2/3); and eukaryotic initiation factor 3 (eIF3) [5]. The m6A modification contributes to different biological functions at the molecular, cellular, and physiological levels, and the dysregulation of m6A modification has been associated with several diseases.

Long non-coding RNAs (lncRNAs), which are abundant and functionally diverse, are over 200 nucleotides in length with limited or no protein-coding capacity [6]. LncRNAs have been implicated in epigenetic modification, mRNA transcription, splicing, stability, translation, and other biological functions [7]. Furthermore, an increasing number of studies have reported that lncRNAs are involved in multiple diseases, such as cancer, diarrhea, and cardiovascular disease. Specific expression patterns of these functional lncRNAs are considered potential disease biomarkers [8]. For instance, the expression of lncRNA *CCDST* has been reported to be significantly downregulated in cervical cancer tissues. It promotes cancer cell migration and angiogenesis by binding to the pro-oncogenic DHX9; in addition, lncRNA *CCDST* functions as a scaffold to promote the formation of MDM2 and DHX9 complexes to accelerate the degradation of DHX9 [9]. Recent studies have demonstrated an important regulatory role of lncRNAs in infectious diarrhea. In addition, host lncRNAs have been identified as a key regulator of host–virus interactions during viral infection [10]. *MPHOSPH9-OT1*, a novel lncRNA identified by high-throughput sequencing in enterotoxin-producing *Escherichia coli* (ETEC)-infected intestinal porcine epithelial cell line-J2 (IPEC-J2), has been reported to induce the secretion of the cytokine CXCL8/IL-8 from IPEC-J2 cells and regulate the host immune response to ETEC infection [11]. Similarly, m6A modifications could affect the functions of lncRNAs in diseases through multiple regulatory mechanisms [12]. The m6A-modified lncRNAs, namely, *ENSSSCG00000048701* and *ENSSSCG00000048785*, have been speculated to be involved in the immune and inflammatory responses of IPEC-J2 cells by regulating their target genes to resist bacteria-induced diarrhea in piglets [13]. However, studies on the mechanisms underlying disease regulation by m6A-modified lncRNAs are limited.

*Clostridium perfringens* type C (*C. perfringens* type C) is highly invasive and can infect domestic animals such as calves, sheep, goats, horses, and piglets, causing necrotizing hemorrhagic enteritis and sudden death syndrome. Moreover, it can infect humans, leading to gastroenteritis-type food poisoning and necrotizing enteritis [14,15]. A human disease called “Darmbrand” enteritis caused by *C. perfringens* type C was related to high mortality in children in Papua New Guinea until a vaccine was invented [16]. The incidence of *C. perfringens* type C infection in piglets is 40 to 50%, with a mortality rate of up to 100%, severely restricting the development of the pig industry and affecting the economic performance of pig production worldwide [17]. The *Clostridium perfringens* β2 (CPB2) toxin is the primary virulence factor produced by *C. perfringens* type C and is associated with necrotizing enterocolitis and enterotoxemia in domestic animals such as pigs, chickens, cattle, and horses [18,19,20,21,22,23]. The CPB2 toxin is highly cytotoxic; the treatment of IPEC-J2 cells with CPB2 toxins has been reported to significantly promote apoptosis and inflammatory responses and disrupt the functions of the intestinal barrier [24,25]. We previously constructed an in vitro cell model of *C. perfringens* type C piglet diarrhea and performed RNA immunoprecipitation sequencing (MeRIP-seq). The methylation and expression of lncRNA *EN_42575* were significantly reduced after the treatment of cells with CPB2 toxins [13]. However, the function of lncRNA *EN_42575* in *C. perfringens* type C-induced diarrhea in piglets and its potential mechanisms warrant further investigation. In this study, we determined the expression pattern of lncRNA *EN_42575*, knocked down and overexpressed lncRNA *EN_42575*, and investigated its regulatory mechanism using a series of cellular assays. The results provide a theoretical basis for exploring the epigenetic perspective of infectious diarrhea in piglets.

## 2. Results

### 2.1. LncRNA EN_42575 Expression Pattern Analysis

**Figure 1:**
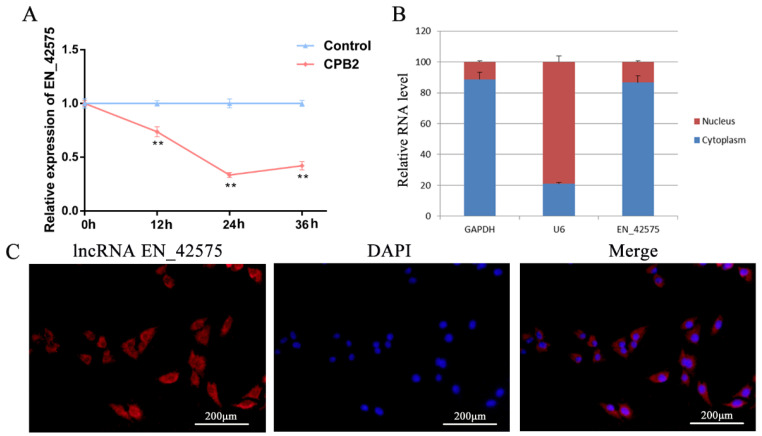
Reduced expression of lncRNA *EN_42575* in IPEC-J2 cells treated with CPB2 toxin. (**A**) This decrease was measured using a qPCR analysis of lncRNA *EN_42575* expression in CPB2-treated IPEC-J2 cells. GAPDH was used as an internal reference. (**B**) The localization of lncRNA *EN_42575* in cells was determined by nuclear/cytoplasmic isolation, with red color representing the ratio of RNA distributed in the nucleus and blue color representing the ratio of RNA distributed in the cytoplasm. U6 and GAPDH served as internal references for nuclear and cytoplasmic RNA, respectively. (**C**) The subcellular localization of lncRNA *EN_42575* in IPEC-J2 cells was detected by RNA–FISH. LncRNA *EN_42575* is stained red (Cy3), and nuclei are stained blue (DAPI). Scale bar = 200 µm. ** *p* < 0.05.

To evaluate the effect of treatment with CPB2 toxins on the expression of lncRNA *EN_42575*, we performed qRT-PCR in IPEC-J2 cells exposed to 20 μg/mL CPB2 toxin for varying durations. The results indicated that CPB2 toxin treatment significantly reduced the expression of lncRNA *EN_42575*; the lowest expression was observed after 24 h of treatment (Figure 1A). Nuclear/cytoplasmic isolation and RNA–FISH analysis revealed that lncRNA *EN_42575* was primarily localized in the cytoplasm (Figure 1B,C). These findings suggested the role of lncRNA *EN_42575* in *C. perfringens* type C-induced diarrhea in piglets.

### 2.2. LncRNA EN_42575 Promotes CPB2-Induced Proliferation of IPEC-J2 Cells

**Figure 2:**
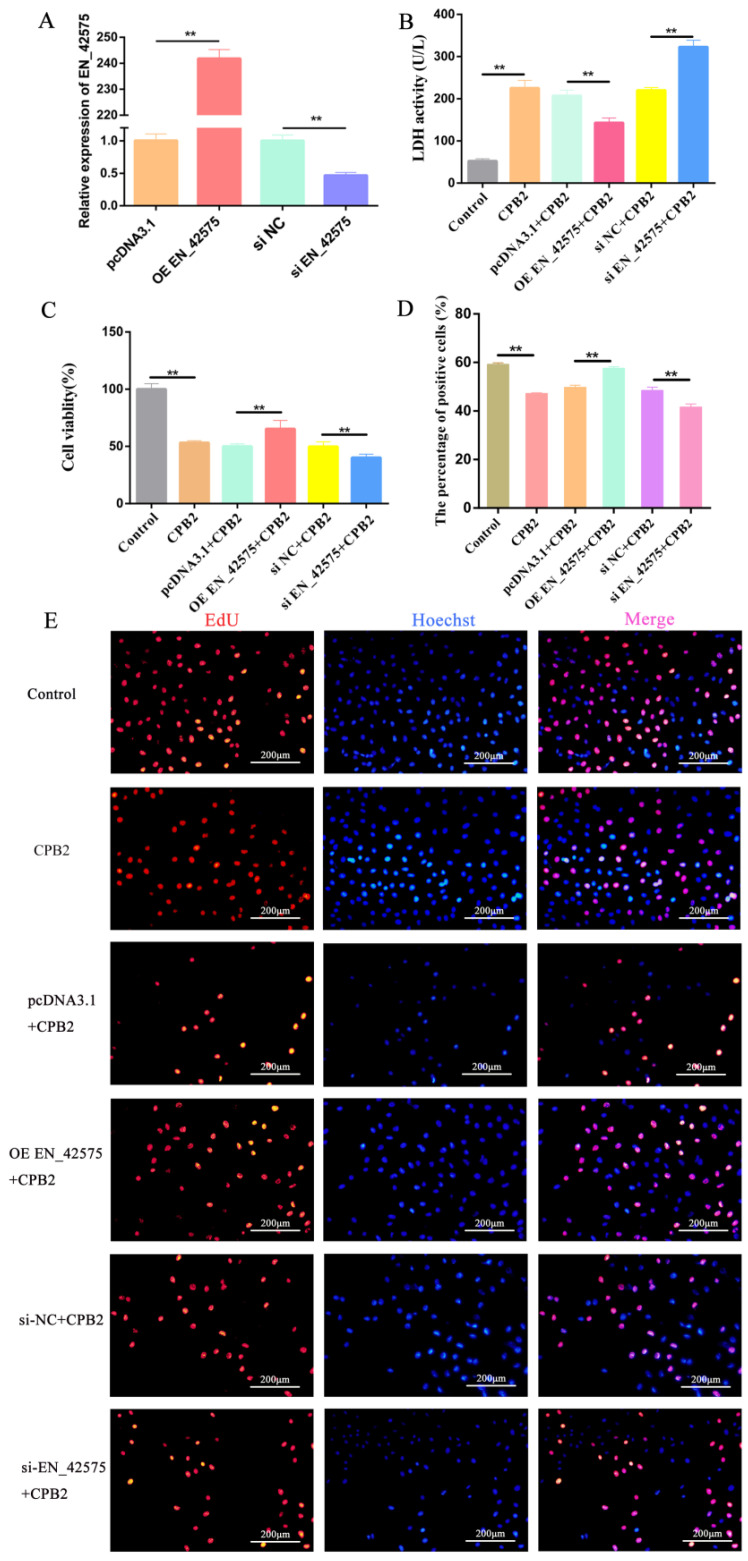
LncRNA *EN_42575* promotes the proliferation of IPEC-J2 cells and alleviates CPB2 toxin-induced cytotoxicity. GAPDH was used as an internal reference. (**A**) LncRNA *EN_42575* overexpression and knockdown efficiency assay. (**B**) LDH activity (absorbance at 450 nm) after transfection of overexpression plasmid OE EN_42575, interfering RNA si EN_42575. (**C**) Viability of IPEC-J2 cells treated with CPB2 toxin after knockdown and overexpression of lncRNA *EN_42575*. (**D**) The proportion of proliferating cells in the EdU assay. (**E**) EdU assay after transfection with overexpression plasmid OE EN_42575 and interfering RNA si EN_42575; cells with DNA replication are stained red with EdU, and nuclei are stained blue with Hoechst3342. Scale bar = 200 µm. ** *p* < 0.05.

We performed qRT-PCR to detect the knockdown and overexpression efficiency of lncRNA *EN_42575*. The results showed that transfection with overexpression plasmid OE EN_42575 markedly increased the expression of lncRNA *EN_42575*, whereas transfection with interfering RNA si EN_42575 significantly decreased its expression (Figure 2A). Next, we detected lactate dehydrogenase (LDH) activity in cell culture supernatants to determine the extent of cell damage and response to CPB2 toxicity. Following the treatment of IPEC-J2 cells with CPB2, the LDH activity was significantly increased, whereas the overexpression of lncRNA *EN_42575* significantly decreased the LDH activity. The knockdown of lncRNA *EN_42575* further increased the LDH activity, indicating that lncRNA *EN_42575* can inhibit cytotoxicity (Figure 2B). Next, the CCK-8 analysis revealed that CPB2 toxin treatment significantly reduced the viability of IPEC-J2 cells. The overexpression of lncRNA *EN_42575* significantly increased CPB2-induced IPEC-J2 cell viability, whereas its knockdown further inhibited the viability of IPEC-J2 cells (Figure 2C). EdU is a cell proliferation marker that directly detects the synthesis of DNA in cells; the proliferating cells appear bright red under fluorescence microscopy. Consistent with the CCK-8 results, the EDU assay demonstrated that the overexpression of lncRNA *EN_42575* promoted cell proliferation, and the knockdown of lncRNA *EN_42575* inhibited cell proliferation (Figure 2D,E). These data suggest that lncRNA *EN_42575* promotes CPB2-induced cell proliferation.

### 2.3. LncRNA EN_42575 Inhibits CPB2 Toxin-Induced Apoptosis and Oxidative Damage

**Figure 3:**
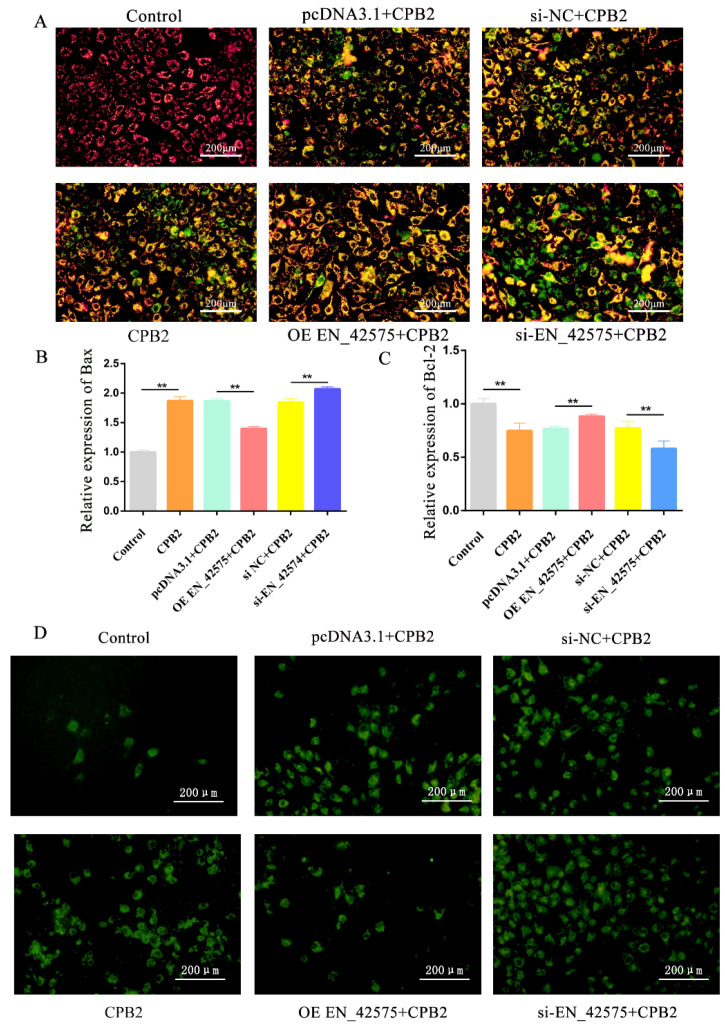
Effects of lncRNA *EN_42575* on CPB2-induced apoptosis and oxidative damage in IPEC-J2 cells. (**A**) Cells were knocked down or overexpressed using lncRNA *EN_42575*, treated with CPB2 toxin for 24 h, and subsequently stained with JC-1. The change in fluorescence from red to green indicated a reduction in the cell membrane potential. (**B**,**C**) The expression of *Bax* and *Bcl-2* was evaluated in CPB2-toxin-treated IPEC-J2 cells following the knockdown or overexpression of lncRNA *EN_42575*. GAPDH was used as an internal reference. (**D**) Effect of lncRNA *EN_42575* on CPB2-toxin-induced ROS levels in IPEC-J2 cells; green fluorescence represents intracellular ROS levels. Scale bar = 200 µm. ** *p* < 0.05.

The mitochondrial transmembrane potential (ΔΨm) decreases during the apoptotic cascade reaction. JC-1, a fluorescent probe, is widely used to detect ∆Ψm. A decrease in the cell membrane potential can be easily detected by a shift in JC-1 fluorescence from red to green. Staining with JC-1 dye revealed red fluorescence in control cells, which gradually shifted to green following the treatment with CPB2 toxins, indicating a decrease in the mitochondrial membrane potential. Moreover, the green fluorescence was reduced after the overexpression of lncRNA *EN_42575* and increased after the knockdown of lncRNA *EN_42575* (Figure 3A). In addition, the overexpression of lncRNA *EN_42575* significantly suppressed CPB2-induced *Bax* (encoding BCL2-associated X, an apoptosis regulator) expression in IPEC-J2 and promoted *Bcl-2* (encoding the BCL2 apoptosis regulator) expression, whereas the opposite trend was observed after the inhibition of lncRNA *EN_42575* (Figure 3B,C). Reactive oxygen species (ROS) in living organisms are produced from oxygen largely via mitochondrial respiration. Their excessive production can lead to oxidative damage in cells. ROS in cells can be detected using the fluorescent probe DCFH-DA, where the green fluorescence represents the level of intracellular reactive oxygen species. The CPB2 treatment of IPEC-J2 cells significantly increased the green fluorescence, indicating an elevated level of ROS. Transfection with the overexpression plasmid OE EN_42575 reduced CPB2 toxin-induced ROS levels, whereas transfection with interfering RNA si EN_42575 further promoted elevated ROS levels (Figure 3D). We therefore concluded that lncRNA *EN_42575* inhibits apoptosis and attenuates CPB2 toxin-induced oxidative damage in IPEC-J2 cells.

### 2.4. LncRNA EN_42575 Is a Target of METTL3

**Figure 4:**
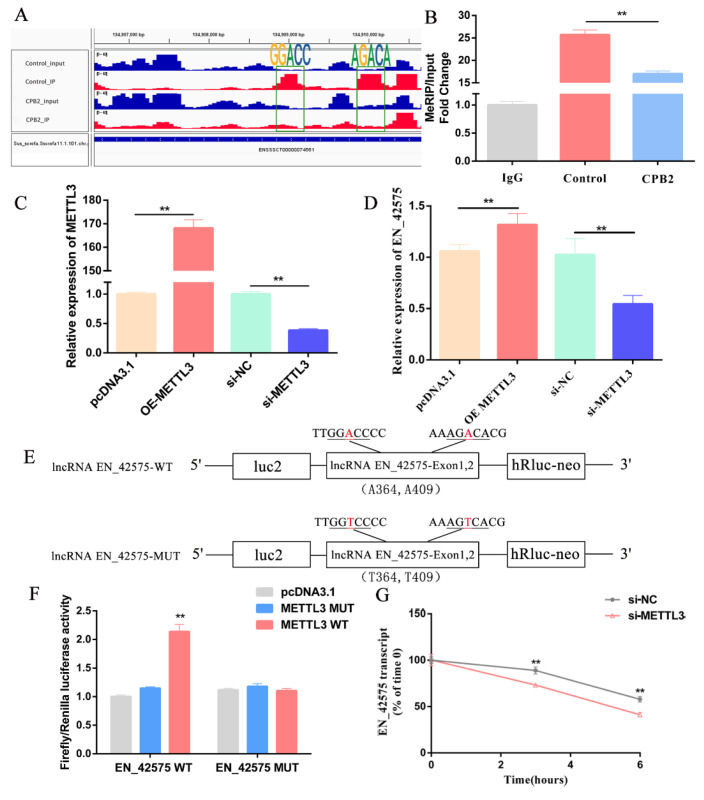
METTL3 positively regulates the expression of lncRNA *EN_42575*. (**A**) The m6A-sequencing detected the m6A abundance in lncRNA *EN_42575* transcripts in the control and CPB2 groups of IPEC-J2 cells. (**B**) The levels of lncRNA *EN_42575* m6A in CPB2-treated IPEC-J2 cells were analyzed by MeRIP-qPCR. (**C**) The efficiency of *METTL3* knockdown and overexpression. (**D**) The lncRNA *EN_42575* expression after *METTL3* knockdown or overexpression. (**E**) LncRNA *EN_42575* WT and MUT (A-to-T mutation) vector. The target fragment (WT, MUT) was loaded into the dual-luciferase reporter vector pmirGLO, with firefly luciferase (*luc2*) as the primary reporter gene and Renilla luciferase (*hRluc-neo*) as the control reporter gene. (**F**) Relative luciferase activity of WT or MUT (A-to-T mutation) lncRNA *EN_42575* in IPEC-J2 cells transfected with pcDNA3.1, METTL3 WT, and MUT METTL3 plasmids. (**G**) The half-life of lncRNA *EN_42575* after *METTL3* inhibition. GAPDH was used as an internal reference. ** *p* < 0.05.

The previous m6A-sequencing results showed that CPB2 toxin treatment significantly reduced the methylation and gene expression of lncRNA *EN_42575* m6A in IPEC-J2 cells (Supplementary Table S1). The m6A-sequencing data of lncRNA *EN_42575* were visualized using Integrative Genomics Viewer software (IGV, version 2.8.13). It was revealed that the two highly enriched specific m6A peaks disappeared after CPB2 toxin treatment (Figure 4A). The MeRIP-qPCR results demonstrated that CPB2 toxin treatment significantly reduced the m6A methylation levels of lncRNA *EN_42575* (Figure 4B).

To investigate the regulation of lncRNA *EN_42575* by methylation-related enzymes, we overexpressed or knocked down METTL3 (Figure 4C). The overexpression of *METTL3* significantly promoted the expression of lncRNA *EN_42575*, whereas its knockdown significantly inhibited the expression of lncRNA *EN_42575* (Figure 4D). To determine whether the m6A modification of lncRNA *EN_42575* is essential for METTL3-mediated gene regulation, we constructed lncRNA *EN_42575*-WT and lncRNA *EN_42575*-MUT vectors (Figure 4E). The forced expression of METTL3-WT significantly increased the luciferase activity of the reporter gene containing the lncRNA *EN_42575*-WT fragment, whereas the overexpression of METTL3-MUT showed no change compared with the control (Figure 4F). The dual-luciferase assay demonstrated that METTL3 regulated the expression of lncRNA *EN_42575* in an m6A-dependent manner. Furthermore, the inhibition of *METTL3* expression significantly reduced the half-life (t1/2) of lncRNA *EN_42575* after blocking the synthesis of cellular RNA with actinomycin D (Figure 4G). These results suggested that METTL3 regulates the expression of lncRNA *EN_42575* through m6A methylation.

### 2.5. Functional Analysis of lncRNA EN_42575 Target Genes

**Figure 5:**
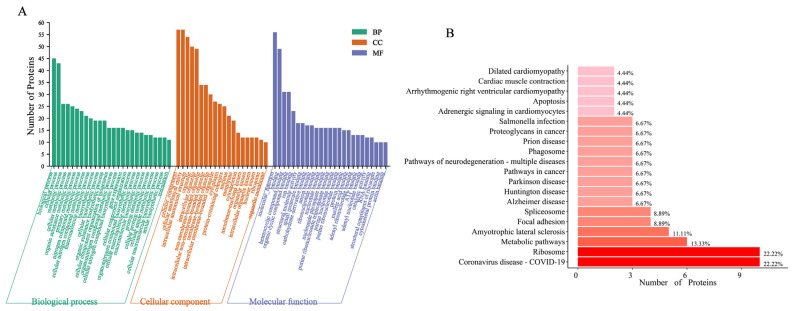
Functional analysis of binding proteins with m6a-modified lncRNA *EN_42575*. Results of the GO enrichment analysis (**A**) and the KEGG signaling pathway analysis (**B**).

To further determine the function of lncRNA *EN_42575* in CPB2-induced IPEC-J2 cells, we performed an RNA pull-down assay of proteins capable of binding to lncRNA *EN_42575*. The mass spectrometry results showed 70 proteins capable of binding to lncRNA *EN_42575* (Supplementary Table S1). The gene ontology (GO) functional enrichment analysis revealed that lncRNA *EN_42575* binding proteins were largely enriched for GO entries such as gene expression, cellular metabolic process, and adenyl ribonucleotide binding (Figure 5A). The Kyoto Encyclopedia of Genes and Genomes (KEGG) pathway enrichment analysis revealed that lncRNA *EN_42575*-binding proteins were enriched in apoptosis, *Salmonella* infection, cancer pathways, and the coronavirus disease (COVID-19) pathway (Figure 5B).

## 3. Discussion

*C. perfringens* type C infection causes extremely high mortality in piglets with diarrhea, severely affecting the economic efficiency of the global pig industry. Currently, the control of piglet diarrhea primarily depends on veterinary drugs and vaccines, which have successfully controlled and reduced the occurrence of diarrhea to a certain extent. However, the long-term use of antibiotics and other veterinary drugs has led to the emergence and prevalence of drug-resistant bacteria, thereby reducing the quality of livestock products. In addition, continuous variations in pathogenic bacteria make the process of vaccine development highly challenging, thus reducing the effective prevention of piglet diarrhea. With the increasing concern for food safety, dependence on drugs to enhance piglets’ resistance to diseases will not be sufficient. The most promising solution is to improve the ability of piglets to resist diarrhea genetically using molecular breeding.

Numerous studies have reported the involvement of dysregulated lncRNAs in the regulation of infectious diarrhea in domestic animals. For instance, porcine lncRNA *FUT3-AS1* regulates *Escherichia coli* F18 susceptibility through histone H4 modification or the miR-212/FUT3 axis [26]. The porcine endemic diarrhea virus infection affects the expression of lncRNAs in the ileum of piglets and is linked to the activation of the immune system in the same region [27]. Similarly, lncRNA *EN-90756* regulates CPB2-induced proliferation and apoptosis in IPEC-J2 cells by affecting the JAK–STAT signaling pathway. In addition, it impacts cellular antiviral capacity by modulating the protein level of MX1 [28]. Previous MeRIP-sequencing studies revealed that m6A-modified lncRNA *EN_42575* was significantly altered in CPB2-group cells [13]. Here, we reported a significantly reduced expression of m6A-modified lncRNA *EN_42575* in IPEC-J2 cells following treatment with the CPB2 toxin. The findings suggested a potential function of lncRNA *EN_42575* in the development of porcine endemic diarrhea associated with *C. perfringens* type C infection.

RRACH is an m6A motif sequence that is highly conserved across numerous species [29]. The use of bioinformatic methods to predict and identify the m6A motif sites of lncRNAs is important to study the metabolism and function of lncRNAs. For example, lncRNAs with the GGACU motif in the mouse cerebral cortex have methylation levels that positively correlate with transcript abundance and are involved in regulating mouse cerebral cortex development [30]. The modification of m6A affects the functions of lncRNAs through several regulatory mechanisms [12]. For example, METTL3 is known to activate the MAPK signaling pathway by regulating m6A modification and lncRNA expression, thereby enhancing the osteogenic differentiation of human adipose-derived stem cells [31]. Similarly, the m6A modification of lncRNA *pncRNA-D* regulates the progression of the HeLa cell cycle by affecting the expression of the *cyclin D1* gene [32]. The m6A demethylase ALKBH5-mediated m6A modification of lncRNA *KCNQ1* overlapping transcript 1 promotes the proliferation, invasion, and metastasis of laryngeal squamous cell carcinoma cells by upregulating *HOXA9* [33]. In non-small-cell lung cancer, METTL3-induced m6A modifications have been reported to enhance the stability and increase the expression of *ABHD11-AS1* transcripts [34]. We found that m6A-modified lncRNA *EN_42575* inhibited CPB2-induced IPEC-J2 cytotoxicity and promoted cell proliferation. Furthermore, m6A-modified lncRNA *EN_42575* attenuated cellular oxidative stress, inhibited the collapse of cellular mitochondrial membrane potential, and suppressed apoptosis in CPB2-induced IPEC-J2. Further experiments demonstrated that METTL3 regulated the expression of lncRNA *EN_42575* in an m6A-dependent manner. Moreover, the inhibition of METTL3 significantly reduced the half-life of lncRNA *EN_42575*.

LncRNAs have been implicated in the regulation of major biological processes. Their mode of function depends on their subcellular localization [35]. LncRNAs in the nucleus can function as activators or repressors of the expression of target genes by directly binding to them. In addition, they regulate gene expression by engaging in histone modifications or recruiting transcription factors [36]. Several nuclear lncRNAs can inhibit tumorigenesis by blocking the activity of PSF proteins to repress proto-oncogene transcription [37,38]. Similarly, cytoplasmic lncRNAs are known to regulate the expression of target genes by interacting with microRNAs, post-transcriptional gene expression, and modulating intracellular signaling pathways [39,40]. The cytoplasmic lncRNA *OCC-1* inhibits the growth of colorectal cancer cells by destabilizing HuR proteins [41]. We reported that lncRNA *EN_42575* is primarily localized in the cytoplasm and regulates CPB2-induced proliferation, apoptosis, and oxidative damage in IPEC-J2 cells. To further investigate the regulatory mechanism of lncRNA *EN_42575*, we performed an RNA pull-down assay that identified 70 proteins interacting with lncRNA *EN_42575*. In addition, GO and KEGG analyses of these proteins revealed them to be primarily involved in gene expression, cellular metabolism, and other processes and enriched in apoptosis, *Salmonella* infection, and cancer pathways. However, the specific regulatory mechanism underlying the functions of lncRNA *EN_42575* has remained largely unknown and warrants further investigation.

## 4. Materials and Methods

### 4.1. Cell Culture and CPB2 Toxin Treatment

IPEC-J2 cells were purchased from BeNa Culture Collection (Beijing, China). The cells were cultured in DMEM high-glucose medium (Hyclone, NY, USA) containing 4500 mg/L glucose with 10% fetal bovine serum (FBS; Gibco, Waltham, MA, USA) and 1% double antibodies (containing penicillin 100 U/mL and streptomycin 100 μg/mL). The cells were maintained at 37 °C with 5% CO_2_. IPEC-J2 cells were treated with 20 mg/mL CPB2 toxin (provided by Gansu Research Center for Swine Production Engineering and Technology) for 24 h to establish an in vitro model of piglet diarrhea.

### 4.2. Cell Transfection

The plasmid pcDNA3.1 was provided by the Gansu Research Center for Swine Production Engineering and Technology. The full-length sequence of 417 bp lncRNA *EN_42575* with *NheI* added at the 5′ end and *XhoI* added at the 3′ end and the full-length sequence of 1743 bp METTL3 with *KpnI* added at the 5′ end and *XhoI* added at the 3′ end were synthesized by GENEWIZ (Suzhou, China). The target fragments were loaded onto the pcDNA3.1 plasmid and named OE EN_42575 and OE METTL3, respectively. The negative control siRNA (si-NC), small-interfering RNA targeting lncRNA *EN_42575* (si EN_42575), and that targeting METTL3 (si-METTL3) were provided by GenePharma (Shanghai, China). The si-NC sequence was 5′-UUCUCCGAACGUGUCACGUTT-3′, the si-METTL3 sequence was 5′-GACGGAUCAUCAAUAAACATT-3′, and the si EN_42575 sequence was 5′-GCCUUAGAUAAAUGCACAATT-3′. IPEC-J2 cells were inoculated on culture plates and, after reaching 70% to 80% confluency, overexpression plasmids or siRNAs were transfected into the cells using Lipofectamine 2000 (Invitrogen, Carlsbad, CA, USA) following the manufacturer’s specifications.

### 4.3. Detection of Lactate Dehydrogenase Activity (LDH)

IPEC-J2 cells (5 × 10^3^ cells/mL) were inoculated in 24-well culture plates with three replicates per group. After plasmid transfection, cell supernatants were collected after treatment with CPB2 toxin. Cytotoxicity was assessed using an LDH cytotoxicity assay kit (Beyotime, Shanghai, China). Finally, the optical density (OD) was measured at 450 nm.

### 4.4. CCK-8 and Δψm Measurements

The cell counting kit-8 (CCK-8) is a rapid and highly sensitive assay kit based on WST-8, which can be reduced by certain dehydrogenases in the mitochondria to produce orange-yellow formazan in the presence of electron-coupled reagents. A high rate of cell proliferation is associated with a darker color. For the CCK-8 assay, IPEC-J2 cells (5 × 10^3^ cells/mL) were planted in 96-well culture plates, ensuring three replicates for each group. After transfection with plasmids and treatment with CPB2 toxins, 10 μL of the CCK-8 solution was added to each well and incubated for 2.5 h. The optical density at 450 nm was measured, and the cell viability was calculated. The Δψm values were measured using the mitochondrial membrane potential assay kit with JC-1 (Beyotime, Shanghai, China). JC-1 is a fluorescent probe widely used to detect ΔΨm. At a high mitochondrial membrane potential, JC-1 aggregates in the mitochondrial matrix and forms polymers (J-aggregates), producing red fluorescence. At low mitochondrial membrane potentials, JC-1 exists as a monomer and produces green fluorescence. A decrease in the cell membrane potential can be easily detected by a shift in the JC-1 fluorescence from red to green. Briefly, IPEC-J2 cells were washed once with PBS solution, and then 1 mL of JC-1 staining working solution was added and incubated for 20 min at 37 °C. Next, the cells were rinsed twice with the JC-1 staining solution, and their fluorescence was observed using a fluorescence microscope (Olympus IX71, Tokyo, Japan).

### 4.5. Reactive Oxygen Species (ROS) Determination

IPEC-J2 (5 × 10^3^ cells/mL) cells were seeded in 24-well plates and underwent transfection with overexpression plasmids (pcDNA3.1 and OE EN_42575) or interfering RNA (si-NC and si EN_42575), followed by exposure to CPB2 toxins for 24 h. Finally, the supernatant was removed, and the cells were washed with PBS. A solution of DCFH-DA (final concentration of 10 µmol/L) in serum-free cell culture medium was added (500 µL), and the cells were incubated for 20 min at 37 °C. Subsequently, the cells were washed thrice with serum-free cell culture medium before being visualized under a fluorescent microscope (Olympus IX71).

### 4.6. Cell Proliferation Assay

IPEC-J2 (5 × 10^3^ cells/mL) cells were grown in 24-well plates and subjected to CPB2 toxin treatment for 24 h. The cells were transfected with overexpression plasmids (pcDNA3.1 and OE EN_42575) or interfering RNA (si-NC and si EN_42575). The proliferation of IPEC-J2 cells was measured using the BeyoClick™ EdU Cell Proliferation kit containing 5-ethynyl-2′-deoxyuridine (EdU) as a proliferation marker (Beyotime). Proliferating cells emitted red fluorescence in the EdU assay due to the incorporation of EdU into the DNA of proliferating cells. Finally, cell proliferation was visualized using fluorescence microscopy (Olympus IX71).

### 4.7. RT-qPCR

The total RNA was extracted from IPEC-J2 cells (1 × 10^7^) using the TRIzol reagent (Invitrogen, CA, USA). IPEC-J2 cytoplasmic and nuclear RNA were obtained according to the instructions provided in the PARIS^TM^ kit (Ambion, Austin, TX, USA). Afterward, the RNA was reverse-transcribed into cDNA using a reverse-transcription kit (Accurate Biotechnology, Changsha, China). Real-time quantitative PCR (qPCR) was performed using the SYBR^®^ Green qPCR Master Mix (Servicebio, Wuhan, China). GAPDH was used as an internal control, and the 2^−∆∆Ct^ method was used to calculate the relative RNA levels [42]. The primer sequences are listed in Table 1.

### 4.8. MeRIP-qPCR

The total RNA (>300 µg) isolated from IPEC-J2 cells was fragmented using the Magna MeRIP™ m6A Kit (Millipore, Billerica, MA, USA), enriched with m6A and IgG antibodies, and purified. Afterward, the RNA was reverse-transcribed, and the enrichment of m6A was determined by qPCR as described in the RT-qPCR section.

### 4.9. Dual-Luciferase Reporter Assay

The sequence containing the m6A-modified fragment of lncRNA *EN_42575* was identified as the wild-type fragment by m6A-sequencing. The A mutation T in the m6A RRACH motif sequence was identified as the mutant fragment. Both of these were synthesized by GENEWIZ and cloned into the pmirGLO dual-luciferase vector (Promega, Madison, WI, USA) and named lncRNA *EN_42575*-WT and lncRNA *EN_42575*-MUT, respectively. The pcDNA3.1 plasmid (named METTL3 WT) carrying the METTL3 cDNA sequence was synthesized by GENEWIZ, and the mutant plasmid (named METTL3 MUT) was obtained by mutating the METTL3 position 1412 A to C. LncRNA *EN_42575*-WT and lncRNA *EN_42575*-MUT were co-transfected with pcDNA3.1, METTL3 WT, or METTL3 MUT in 293T cells. The relative luciferase activity was determined using a dual-luciferase reporter assay system (Promega) according to the manufacturer’s instructions. Normalization was performed using Renilla luciferase to evaluate the dual-luciferase reporter data.

### 4.10. Single-RNA Fluorescence In Situ Hybridization (FISH)

For FISH assays, IPEC-J2 cells were grown in 24-well plates and cultured for 24 h. Following fixation and permeabilization, IPEC-J2 cells were hybridized with 10 μM cy3-labeled lncRNA *EN_42575*. The nuclei were stained with 6-diamidino-2-phenylindole (DAPI). After washing the cells with PBS, images were acquired using a camera fitted to a fluorescence microscope (Olympus IX71).

### 4.11. RNA Stability

To determine the stability of the RNA, IPEC-J2 cells were treated with 5 μg/mL actinomycin D (Sigma, St. Louis, MO, USA) for 0, 3, and 6 h. Cells were collected, and RNA samples were extracted for reverse transcription. Quantitative PCR was performed to detect the levels of RNA transcripts.

### 4.12. RNA Pull-Down and Mass Spectroscopy Analyses

Biotin-labeled lncRNA *EN_42575* and antisense RNA were transcribed in vitro using the mMESSAGEmMACHINE^TM^ T7 kit (Invitrogen, Carlsbad, CA, USA). The primers used are listed in Supplementary Table S1. The transcribed RNAs were purified using the RNeasy Mini Kit (QIAGEN, Valencia, CA, USA) following the manufacturer’s instructions. The RNA pull-down assay was performed by incubating biotin-labeled RNA with IPEC-J2 cell lysate using the RNA-protein pull-down kit (Thermo, Waltham, MA, USA). The mass spectrometry analysis of the proteins bound to lncRNA *EN_42575* was performed by FitGene Biotechnology (Guangzhou, China).

### 4.13. Statistical Analysis

SPSS v.21 software was used for statistical analysis. The data are presented as mean ± standard deviation (SD). Statistical analyses were performed using Student’s *t*-tests and one-way analysis of variance (ANOVA), depending on the purpose and type of data. A *p*-value < 0.05 was considered significant.

## 5. Conclusions

In conclusion, we found that the m6A modification of lncRNA *EN_42575* was significantly downregulated following CPB2 treatment. The overexpression of lncRNA *EN_42575* reduced cytotoxicity, promoted cell proliferation, and inhibited apoptosis and oxidative damage, whereas its knockdown produced a reverse effect. METTL3 regulated the expression of lncRNA *EN_42575* in an m6a-dependent manner. These findings highlighted the critical function of m6A-modified lncRNAs in CPB2 toxin-induced diarrhea in piglets, thus revealing a novel mechanism for the regulation of lncRNAs. This study provides a new avenue for the future investigation of the epigenetic regulation patterns of RNAs.

## Figures and Tables

**Table 1:** Details of primer sequences used for MeRIP-qPCR and RT-qPCR.

Gene Name/Id		Primer Sequence (5′-3′)	Product Length	Type
ENSSSCG00000042575	Forward	TGAATCAGCAGATACGGGCA	82	MeRIP-qPCR/RT-qPCR
	Reverse	GAAACTTGTACGGGCATCCA		
Bax	Forward	GCTGACGGCAACTTCAACTG	202	RT-qPCR
	Reverse	GCGTCCCAAAGTAGGAGAGG		
Bcl-2	Forward	GGTGAACTGGGGGAGGATTG	130	RT-qPCR
	Reverse	GTGCCGGTTCAGGTACTCAG		
METTL3	Forward	CCACTTCTGGTGGCCCTAAG	104	RT-qPCR
	Reverse	CGCCAGATCAGAAAGGTGGT		
GAPDH	Forward	AGTATGATTCCACCCACGGC	139	RT-qPCR
	Reverse	TACGTAGCACCAGCATCACC		
U6	Forward	TTATGGGTCCTAGCCTGAC	224	RT-qPCR
	Reverse	CACTATTGCGGGTCTGC		

## Data Availability

Not applicable.

## Supplementary Materials

The following supporting information can be downloaded at: https://www.mdpi.com/article/10.3390/ijms24065725/s1.
